# Assessing Mobile Phone Access and Perceptions for Texting-Based mHealth Interventions Among Expectant Mothers and Child Caregivers in Remote Regions of Northern Kenya: A Survey-Based Descriptive Study

**DOI:** 10.2196/publichealth.5386

**Published:** 2017-01-30

**Authors:** Abdul Momin Kazi, Jason-Louis Carmichael, Galgallo Waqo Hapanna, Patrick Gikaria Wangoo, Sarah Karanja, Denis Wanyama, Samuel Opondo Muhula, Lennie Bazira Kyomuhangi, Mores Loolpapit, Gilbert Bwire Wangalwa, Koki Kinagwi, Richard Todd Lester

**Affiliations:** ^1^ Experimental Medicine Department of Medicine University of British Columbia Vancouver, BC Canada; ^2^ Division of Maternal and Child Health Paediatrics and Child Health The Aga Khan University Karachi Pakistan; ^3^ WelTel International mHealth Society Vancouver, BC Canada; ^4^ AMREF Health Africa Nairobi Kenya; ^5^ Division of Infectious Diseases Faculty of Medicine University of British Columbia Vancouver, BC Canada

**Keywords:** mobile health, text messaging, prenatal care, immunization, Kenya

## Abstract

**Background:**

With a dramatic increase in mobile phone use in low- and middle-income countries, mobile health (mHealth) has great potential to connect health care services directly to participants enrolled and improve engagement of care. Rural and remote global settings may pose both significant challenges and opportunities.

**Objective:**

The objective of our study was to understand the demographics, phone usage and ownership characteristics, and feasibility among patients in rural and remote areas of Kenya of having text messaging (short messaging service, SMS)-based mHealth intervention for improvements in antenatal care attendance and routine immunization among children in Northern Kenya.

**Methods:**

A survey-based descriptive study was conducted between October 2014 and February 2015 at 8 health facilities in Northern Kenya as part of a program to scale up an mHealth service in rural and remote regions. The study was conducted at 6 government health facilities in Isiolo, Marsabit, and Samburu counties in remote and northern arid lands (NAL). Two less remote health facilities in Laikipia and Meru counties in more populated central highlands were included as comparison sites.

**Results:**

A total of 284 participants were surveyed; 63.4% (180/284) were from NAL clinics, whereas 36.6% (104/284) were from adjacent central highland clinics. In the NAL, almost half (48.8%, 88/180) reported no formal education and 24.4% (44/180) self-identified as nomads. The majority of participants from both regions had access to mobile phone: 99.0% (103/104) of participants from central highlands and 82.1% (147/180) of participants from NAL. Among those who had access to a phone, there were significant differences in network challenges and technology literacy between the 2 regions. However, there was no significant difference in the proportion of participants from NAL and central highlands who indicated that they would like to receive a weekly SMS text message from their health care provider (90.0% vs 95.0%; *P=*.52). Overall, 92.0% (230/250) of participants who had access to a telephone said that they would like to receive a weekly SMS text message from their health care provider. Most phone users already spent the equivalent of 626 SMS text messages on mobile credit for personal use.

**Conclusions:**

Despite the remoteness of northern Kenya’s NAL, the results indicate that the majority of pregnant women or care givers attending the maternal, newborn, and child health clinics have access to mobile phone and would like to receive text messages from their health care provider. mHealth programs, if designed appropriately for these settings, may be an innovative way for engaging women in care for improved maternal and newborn child health outcomes in order to achieve sustainable development goals.

## Introduction

It has been widely documented that routine antenatal care (ANC) visits is one of the most effective methods in reducing maternal and child mortality and morbidity. Despite calls by the World Health Organization (WHO) for a minimum of 4 ANC visits, two-thirds of women living in Africa are able to attend ANC clinic only once during their gestational period [1,2]. It is estimated that 287,000 maternal deaths occur globally each year due to preventable complications during pregnancy and childbirth, 95% of which occur in sub-Saharan Africa and Southern Asia [3,4]. Furthermore, approximately 240 women per 100,000 live births die in low-income countries compared with only 16 women per 100,000 live births in high-income countries. African women experience 24 times the risk of intrapartum stillbirth compared with women in high-income settings [5-7]. Globally, there are around 3 million neonatal deaths and 2.65 million stillbirths each year [7]. In Kenya, the annual maternal mortality rate is 488 per 100,000 live births, of which only 47% of women are able to attend all 4 recommended ANC visits and the majority of them are from urban areas [8-10]. A further decline is seen in the remote rural areas of Kenya such as the northern and arid lands (NAL) whereby only 40% of pregnant women attend 4 or more ANC visits [8].

Routine immunization among children, on the contrary, is one of the most effective public health interventions of the 20th century. It has dramatically reduced global child morbidity and mortality [11]. Despite this, an estimated 19.3 million children did not receive vaccination for diphtheria, pertussis, and tetanus (DPT3) in 2010, one-third of which live in Africa, resulting in approximately 1.5 million preventable deaths every year [12]. In Kenya, approximately 52 infants per 100, 000 die every year, with only 77% receiving routine immunizations [8-10]. New and innovative strategies are required to improve ANC attendance among pregnant women and to increase routine immunization coverage among children.

Mobile health (mHealth) is defined as mobile communication technologies to support health care [13,14]. Currently, there are approximately 7 billion mobile phone subscribers globally, 89% of which are in low- and middle-income countries (LMICs) [15,16]. In Kenya, for example, mobile phone penetration was 77% in 2012, with SMS text messaging (short messaging service, SMS)-based communication on the rise [17]. The recent surge in mobile phone penetration alongside reduced cost has improved the feasibility of mHealth programs in LMICs [18]. Available data outlines mHealth’s potential and ability to connect health care services to pregnant women and mothers by passing different hurdles that would otherwise keep various health services inaccessible due to cost, distance, and accessibility [18,19]. SMS text messaging–based mHealth interventions can play a significant role in ensuring that the expectant mothers are followed throughout the continuum of care till completion of routine immunization coverage of their child [19].

Existing literature on mobile phone use in Kenya focuses on mobile phone penetration, trends in uptake of mobile communication services, corporate market share, mobile consumer-based services available, ANC visits improvement, SMS text message reminders and conditional cash transfer to improve routine immunization coverage, potential for mHealth interventions based on phone ownership and penetration, and engagement of care [20-26]. Few studies, however, examine the demographic characteristics of mobile phone users, their usage patterns, ownership, and mobile phones’ potential for improving engagement with health care providers and access to health services, specifically in a remote and rural population.

There is a vast difference in health indicators for NAL compared with rest of the Kenya, particularly for health-seeking behavior, maternal and child mortality, and immunization coverage. In this study, we aimed to assess the feasibility of implementing an SMS text messaging–based mHealth intervention intended to connect expectant mothers and child caregivers with their health care providers in rural and remote regions of Kenya. Our objectives were to examine region-based differences in mobile phone access and mHealth perceptions among visitors to antenatal and routine immunizations clinics in 8 regions of northern Kenya.

## Methods

**Figure 1:**
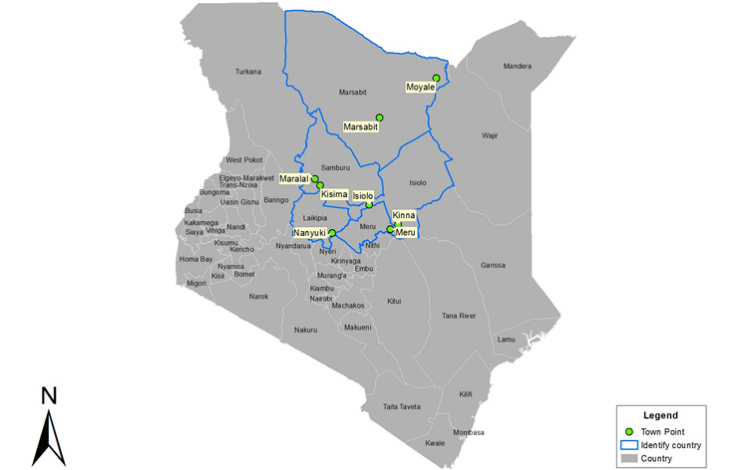
Geographic distribution of the eight sites participating in baseline survey in Kenya.

Between October 13, 2014, and February 20, 2015, participants were recruited from antenatal and routine immunization clinics at 8 health facilities in northern Kenya as part of a Grand Challenges Canada phase 2 “transition to scale” and Amref Health Africa as part of government of Kenya–led AphiaPlus/Marisha program for the health strengthening in the region [26]. Six participating government health facilities, 2 each in Isiolo, Marsabit, and Samburu counties were located in Kenya’s remote NAL, and 2 clinics each were located in Laikipia and Meru counties, which are less remote and more densely surrounding northern region (central highlands). The study received clearance from Amref Health Africa’s Ethics and Scientific Review Committee on September 1, 2014. Figure 1 is a graphic depicting distribution of all 8 sites.

The study questionnaires included sections on patient demographics, mobile phone usage patterns, the feasibility of an SMS text messaging–based mHealth intervention, as well as accessibility to primary health centers. Because the proposed mHealth intervention (WelTel) used weekly SMS text messages and allowed for shared phone access, questions related to this were included [27]. Inclusion criteria comprised pregnant women visiting the antenatal clinic, caregivers attending the routine immunizations clinics for children, and the ability to provide informed consent. The formal sample size calculation was not done; however, a target of 280 completed surveys was set. Consecutive convenience sampling was used to recruit patients whereby health care workers at the point of triage referred patients to study staff as patients arrived for their scheduled ANC and routine immunization appointments.

Structured questionnaires were administered in either English or Kiswahili depending on a patient’s preference and proficiency with English by trained study staff having command over both languages. All participants included in the study were asked to sign the informed consent, and thumb impression was taken from those who were illiterate or could not sign. Questionnaires were administered in a private place to ensure patient confidentiality and reduce the likelihood of response bias. No incentive was provided for participating in this study.

Prior to the study, questionnaires were developed and underwent forward and backward translation to ensure semantic consistency, and were piloted at one of the field sites before being rolled out. The research staff received 2-day training on research ethics, providing informed consent, administering the study questionnaire, and the study protocols to be followed throughout the course of the study. Continuous monitoring by study coordinators occurred throughout the study to ensure that data were collected as per the study protocol. All data was double entered into an Access program and analyzed using IBM SPSS Statistics version 23, with a level of significance in reference to a 2-tailed, type 1 error set as <0.05. Univariate analysis was performed using the chi-square test or Fisher exact test for dichotomous variables and the Mann-Whitney test for continuous variables.

## Results

Distribution of the respondents among study sites and population demographics at the study sites are shown in Table 1. Overall, 284 participants completed the survey from the 8 health facilities. Specifically, 63.4% (180/284) from the NAL clinics (Samburu, Isiolo, and Marsabit counties), and 36.6% (104/284) patients participated from central highlands (Meru and Laikipia counties). Overall, 98.6% (280/284) of the participants were females, and the median age of participants was 26 years (interquartile range, IQR 23-30 years). Forty-four percent (125/284) of participants were pregnant women visiting the ANC clinic, whereas 56.0% (159/284) were caregivers of infants attending the immunization clinic for routine immunizations.

**Table 1:** Baseline characteristics of participants, by region.

Characteristics		Northern arid land (n=180)	Adjacent site (n=104)	Total (N=284)
**Visited department, n (%)**				
	Antenatal care	72 (40.0)	53 (51.0)	125 (44.0)
	Immunization	108 (60.0)	51 (49.0)	159 (56.0)
**Facilities, n (%)**				
	Isiolo district hospital	19 (11.0)		19 (6.7)
	Kinna health center	31 (17.2)		31 (10.9)
	Maralal district hospital	30 (16.7)		30 (10.6)
	Kisima model health center	58 (32.2)		58 (20.4)
	Marsabit districts hospital	21 (11.7)		21 (7.4)
	Moyale district hospital	21 (11.7)		21 (7.4)
	Meru level 5 hospital		53 (51.0)	53 (18.7)
	Nanyuki reference hospital		51 (49.0)	51 (18.0)
Age, median (IQR^a^)		27 (23-30)	24 (22-29)	26 (23-30)
**Gender, n (%)**				
	Female	176 (97.8)	104 (100.0)	280 (98.6)
	Male	4 (2.2)		
**Marital status, n (%)**				
	Single	8 (4.4)	15 (14.4)	23 (8.1)
	Married	164 (91.1)	84 (80.8)	248 (87.3)
	Separated, widowed, or dating	8 (4.4)	5 (4.8)	13 (4.6)
Number of children, median (IQR)		2 (1-4)	1 (1-1)	2 (1-3)
Nomadic lifestyle, n (%)		44 (24.4)	7 (6.7)	51 (18.0)
**Formal education, n (%)**				
	None	88 (48.9)	2 (1.9)	90 (31.7)
	Primary	57 (31.7)	30 (28.8)	87 (30.6)
	Secondary	24 (13.3)	41 (39.4)	65 (22.9)
	College or University	11 (6.1)	31 (29.8)	42 (14.8)
**Religion, n (%)**				
	Christian	97 (53.9)	99 (95.2)	196 (69.0)
	Islam	76 (42.2)	3 (2.9)	79 (27.8)
	Others	7 (3.9)	2 (1.9)	9 (3.1)
Formally employed, n (%)		93 (51.7)	50 (48.0)	143 (50.3)
**Preferred spoken language, n (%)**				
	Swahili	77 (42.8)	48 (46.1)	125 (44.0)
	Kiborana	54 (30.0)		54 (19.0)
	Samburu	38 (211)		38 (13.3)
	Kimeru	3 (1.6)	25(24.0)	28 (9.9)
	English	4 (2.2)	18 (17.3)	22 (7.7)
	Other	4 (2.2)	13 (12.5)	17 (6.0)
Distance from health center in minutes, mean (min-max)		64 (2-300, n=166)	35 (5-300, n=102)	53 (2-300, n=268)

^a^IQR: interquartile range.

When compared with participants from central highlands, a higher proportion of participants from the NAL were more likely to be married (91.1% vs 80.8%; *P*=.01) and self-identify as nomadic (24.4% vs 6.7%; *P*<.001). There were large differences in the proportion of participants who had received formal education: 48.9 % (88/180) of respondents from NAL reported having no formal education compared with 1.9% (2/104) from central highlands (*P*<.01). Similarly, a much higher proportion of participants from the central highlands clinics could understand (98.0% vs 52.2%; *P*<.001) and read (91.3% vs 46.7%; *P*<.001) common Swahili words (“mambo,” “sawa,” and “shida”) when compared with participants from NAL. Fifty percent (142/284) of the participants were employed of which the most common occupation was cited as a business.

Overall, 74.3% of participants (211/284) owned a mobile phone across all sites. However, a significantly higher proportion of participants from central highlands owned their phones when compared with participants from NAL (94.2% vs 62.8%; *P*<.001). Out of those who did not own a mobile phone 53.4% (39/73) did have shared access to a mobile phone with (83.3% vs 51.5%; *P*<.001) citing shared access in central highlands as compared to NAL. This indicated that the majority of participants from both the central highlands and NAL had some access to a mobile phone (99.0% vs 82.1%, *P*<.001).

A higher proportion of those from NAL were more likely to experience network challenges (27.1% vs 14.6%; *P*<.001). However, 92.1% (35/38) in NAL and 93.3% (14/15) at adjacent site participants were able to overcome the network coverage issues and only 9.2% (26/284) reported of a problem with keeping their phone battery charged overall. Further participants from NAL were less likely to be able to send an SMS text message (55.0% vs 95.1%; *P*<.001) when compared with participants from central highlands. Of those who were not able to send an SMS text message, 93.7% (59/63) in NAL and 80.0% (4/5) in adjacent sites mentioned that a trusted individual could respond on their behalf. Tables 2 and 3 show phone ownership and usage characteristics and SMS text messaging and mobile phone related outcomes.

Despite regional differences, there was no significant difference in the proportion of participants from NAL and central highlands, who indicated that they would like to receive a weekly SMS text message from their health care provider (95.0% vs 97.0%; *P*=.52). Overall, more than 91.6% (230/251) of participants who had access to a mobile phone indicated that they would like to receive a weekly SMS text message from their health care provider. Of these, 51.2% (124/242) preferred SMS text message as their mode of communication, whereas 48.8% (118/242) favored a phone call. Regional variances existed. Participants (78.2% vs 31.9%; *P*<.001) in central highlands preferred SMS text message compared with NAL; however, participants (68.1% vs 21.8%; *P*<.001) in NAL preferred phone calls as opposed to central highlands. Further, 62.5% (157/251) of responders preferred receiving SMS text messages in Swahili. Moreover, 49.4% (124/251) cited no time-of-day preference for receiving SMS text messages, and 40.6% (102/251) responded in favor of receiving 1 SMS text message per week. The average amount of money spent on a mobile phone by patients in the survey every month was 664 KSH (US $6.57), equivalent to about 170 minutes of talk time or more than 626 SMS text messages. The mean distance of the participants’ home from health care center was 53 minutes (n=268/284).

**Table 2:** Regional differences in mobile phone access.

Characteristic		Northern arid land (n=180)	Adjacent region (n=104)	Total (n=284)	*P* value
**Telephone access^a^, n (%)**					<.001
	Access to telephone	147 (81.7)	103 (99.0)	250 (88.0)	
	No access to telephone	32 (17.8)	1 (1.0)	33 (11.6)	
	Own phone	113 (62.8)	98 (94.2)	211 (74.3)	
	Shared phone	34 (18.9)	5 (4.8)	39 (13.7)	
	No telephone access	32 (17.8)	1 (1.0)	33 (11.6)	

^a^Missing data: 1 northern arid land.

**Table 3:** Regional differences in SMS text messaging– and phone-related outcomes, among those who had access to mobile phone.

Characteristics		Northern arid land (n=147)	Surrounding northern region (n=103)	Total (n=250)	*P* value
**Experienced network challenges^a^, n (%)**					<.001
	No	102 (72.9)	88 (85.4)	190 (76.0)	
	Yes	38 (27.1)	15 (14.6)	53 (21.2)	
**Problem keeping a phone charged^b^, n (%)**					.41
	No	124 (87.9)	94 (91.3)	218 (89.3)	
	Yes	17 (12.1)	9 (8.7)	26 (10.7)	
**Preferred mode of communication^c^, n (%)**					<.001
	Text message	45 (31.9)	79 (78.2)	124 (49.6)	
	Telephone call	96 (68.1)	22 (21.8)	118 (47.2)	
**Are able to send an SMS text message^d^, n (%)**					<.001
	Yes	77 (55.0)	97 (95.1)	174 (69.6)	
	No	63 (45.0)	5 (4.9)	68 (27.2)	
**Would like to receive a weekly SMS text message from HCP^e^, n (%)**					.53
	Yes	132 (95.0)	98 (97.0)	230 (92.0)	
	No	7 (5.0)	3 (3.0)	10 (4.0)	

^a^Missing data: 7 northern arid lands.

^b^Missing data: 6 northern arid lands.

^c^Missing data: 6 northern arid lands and 2 surrounding northern regions.

^d^Missing data: 7 northern arid lands and 1 surrounding northern region.

^e^Missing data: 8 northern arid lands and 2 surrounding northern region.

## Discussion

### Principal Findings

There is a significant potential for using mHealth communication to strengthen health services for maternal, newborn, and child health (MNCH) in remote underserved areas, where access to health services can otherwise be extremely limited. This study described the patients’ access to mobile phones in the health context in a remote rural population with a high proportion of pastoralists and where maternal and child morbidity and mortality are disproportionately high. The majority of women and caregivers attending ANC and routine immunization clinics had access to a mobile phone—82.1% in NAL and 99.0% in the more populated highlands—and overall 92.0% of the participants indicated that they would like to receive weekly SMS text messages. The millennium development goals for maternal child health have not been successfully achieved by 2015 at multiple LMICs including Kenya. There is a broad consensus that mHealth can play an essential role in improving the use of maternal and child health services, which may ultimately support in decreasing morbidity and mortality toward achieving a sustainable development goal of less than 70 maternal deaths per 100,000 live births globally by 2030 [28].

The mobile phone ownership and access detailed in this study were similar to those in other studies conducted among women attending antenatal care at primary health centers in LMIC settings [29-31]. There was a significantly high proportion of participants from central highlands who owned mobile phones compared with NAL (94.2% vs 62.8%). Similarly, 69.6% of the participants in central highlands were able to send SMS text messages compared with only 55.0% in NAL. Although around half of the population in NAL was not able to send the SMS text messages, a vast majority among them, 92.6%, trusted someone who could send a message in their place. Social innovations such as utilizing shared phone access, combined with technological innovations, are likely important to maximize the reach to the majority of the population who may benefit from this support. In addition, if the messaging can be kept simple, there may be an eagerness for women with even minimal literacy to learn some basic texting. This indicates a significant opportunity for mHealth-based interventions to strengthen MNCH programs in the region considered among the most remote places in the world.

Throughout the region, there was high acceptance among the participants to receive SMS text messages (92%) from health care providers inquiring about individual’s health. Importantly, 68.1% of NAL responder’s and 21.8% in the adjacent sites preferred phone call versus text messaging respectively. This was similar to a study conducted by Cormick et al in Argentina, where respondents from rural pollution compared with the urban setup preferred talking on a phone versus texting [29]. In addition to literacy, women might be wary of potential risk brought about by stigma if a family member or friend became privy to a patient’s medical condition through SMS text messaging. This suggests that the content of SMS text messages be carefully considered, and before implementing an SMS text message–based mHealth intervention, some advocacy of the intervention and teaching of SMS content and use might be required for improved acceptability. Combining phone calls with SMS text messages may also overcome some of the barriers.

A number of SMS text message–based interventions have been quite effective in different programs, particularly in treatment adherence, smoke cessation, health care appointment attendance, antenatal care attendance, and compliance for immunization [29-34]. Also, adding incentives to SMS text messages have shown a positive association. However, there is a cost implication for scaling up this model at the country level [25,29]. The major focus up till now has been on SMS text message as reminders or educational messages for improvement in behavioral change related to MNCH in LMIC settings [24,32,33]. The WelTel service proposed in this study population utilized two-way SMS text messages that checked women’s status and provided an opportunity for triage of any health issues they had on a standardized basis. Given the mobile phone access and acceptability in both the ANC and post-delivery immunization population, there is potential to maximized interventions that can be delivered along the continuum of care from early pregnancy and through childbirth to the newborn period, through the immunization period in the first years of life. Two-way SMS text message–based models compared with one-way SMS text message or a combination of automated voice message or a phone call needs to be assessed [34]. This is essential for understanding mHealth role for an improvement in health-seeking and vaccine coverage behavior in LMIC setup [30,32-35].

A significant hindrance for SMS text message–based mHealth interventions is the level of education and literacy in some populations. Compared with participants from NAL there was a statistically significant difference in participants in central highlands, who had received formal education and could understand and write common Swahili words (Mambo, Sawa, and Shida). There is a limitation of sending only 160 characters as SMS text messages, which becomes even lesser if translated into another language. However, these restrictions might help in making the message simple and understandable, especially to low-literacy population [33]. One example is WelTel-based model in Kenya, in which single-word text “Mambo” in Swahili (“How are you?” in English) was sent once a week to patients starting antiretroviral therapy who were asked to respond as “Sawa” or “Shida” (“fine or not fine”). In case of receiving “not fine” message, the health care providers called the patients to inquire regarding their status. This simple engagement in mHealth model helped improve antiretroviral therapy adherence and viral suppression [26]. Similar engagement in care model can be used among women and caregivers attending ANC and immunization clinics to bring the needed behavioral change for improvement in the ANC attendance by pregnant women to least 4 visits during pregnancy and vaccination coverage to 90% as recommended by WHO.

Although people in remote settings may stand the most to benefit from mHealth, there are several unique challenges. Mobile phone network challenges were encountered only by one-fifth of the participants, more likely in the remote regions, and among them, more than 90% were able to overcome the network coverage problem. Further, less than 10% of participants reported challenges with keeping their phones charged. Similarly, most small shops had a small fee for charging services. Home solar charging units are becoming increasingly popular. Our study supports the evidence demonstrating a rapid increase in mobile phone access among pregnant women and caregivers of children eligible for routine immunization both globally and in Africa, including in some of the most remote regions of Africa such as northern Kenya. Overall, however, these results are encouraging and show the potential for SMS text messaging–based mHealth interventions in improving communication of health care providers with patients and clinic attendants to improve engagement in care and adherence.

### Limitations

There are a number of limitations to this study. The study was limited to patients enrolled in MNCH services in selected health facilities in northern Kenya’s NAL, and the results may not be generalized to women who never attended the clinic for MNCH care. This may be better assessed by a community-based survey. This study was also of limited sample size and used a convenient sampling method. In this case, a compromise was made to allow for rapid evaluation of the intended population for critical phone-related health characteristics that could assist implementation of an already funded project. Nonetheless, this survey provides new information and insight into the potential for mHealth programs in MNCH in remote pastoral regions. Additional studies are encouraged to evaluate the cost-effectiveness, infrastructure, and human resources required to implement behavioral change through simple mobile phone and SMS text messaging–based interventions.

### Conclusions

In conclusion, despite remoteness, the majority of the pregnant women and caregivers visiting the antenatal care and routine immunization facilities had access to mobile phones and showed high acceptability for weekly SMS text messages in northern Kenya. Mobile phone access is not, however, evenly distributed, and some populations have lower levels of literacy or access to mobile phones or reliable network connections. Using low-cost interventions such as SMS text messaging with simple language or by enabling voice calling that is locally appropriate may help overcome some of these challenges. It is important for programs aiming to reach those who typically have least health care access to understand the details around phone access and ownership. This study indicates that people living in remote regions, with often poor access to quality health services, may also be able to harness the mobile phone revolution and be beneficiaries of mHealth innovations.

## Abbreviations

ANC: antenatal care
LMICs: low- and middle-income countries
MNCH: maternal, newborn, and child health
NAL: northern arid lands
SMS: short messaging service
